# Laryngocele after Subtotal Laryngectomy

**Published:** 2018-09

**Authors:** Mohamed Dhaha, Souheil Jbali, Sawssen Dhambri, Maroua Mahjoub, Slim Touati, Said Gritli

**Affiliations:** 1 *Department of Head and Neck Surgery, Salah Azaez oncology Institute, Tunis, Tunisia.*

**Keywords:** Cricohyoidoepiglottopexy, Laryngeal cancer, Laryngocele, Subtotal laryngectomy

## Abstract

**Introduction:**

Laryngocele is an air-filled dilatation of the laryngeal saccule that extends upward within the false vocal folds. Different etiologies lead to laryngocele congenital malformation, weakness of the laryngeal tissues and increased intralaryngeal pressure. Laryngocele may be a secondary iatrogenic complication following subtotal laryngectomy.

**Case Report:**

We report the case of a 61-year-old patient who presented an external laryngomucocele 8 years after a supracricoid partial laryngectomy with cricohyoidoepiglottopexy (SCPL-CHEP). We focus on the clinical aspects and therapeutic attitude, then discuss the physiopathological conditions that could generate this late complication.

**Conclusion:**

Laryngocele after subtotal laryngectomy should be considered a late iatrogenic complication. Histological examination is necessary after surgical management of laryngocele, as the association with cancer is frequent.

## Introduction

The term laryngocele refers to the benign dilatation of the laryngeal saccule which communicates with the laryngeal lumen. The term was first introduced by Virchow in 1867. Depending on their relation to the thyroid membrane, we distinguish between internal, external, and mixed laryngoceles (1). In fact, laryngocele can extend internally into the airways or externally through the thyroid membrane, and can secondarily be filled with mucus of the glandular secretion (laryngomucocele) or become infected (laryngopyocele). Laryngocele can be either asymptomatic or demonstrate several clinical aspects such as neck swelling, cough, stridor and dysphonia (2). Increased intralaryngeal pressure, weakness of the laryngeal tissues and congenital predisposition are classically the main conditions leading to genesis of laryngocele. However, these cystic aerated dilatations of the laryngeal saccule may appear following laryngeal surgery. Only a few studies have described iatrogenic cases of laryngocele following subtotal laryngectomy (3–5). Head and neck surgeons should be aware of this particular iatrogenic complication, its clinical expression and the physiopathological conditions leading to its creation. We report the case of a 61-year old patient who presented laryngomucocele as a late complication of supracricoid partial laryngectomy with cricohyoidoepiglottopexy (SCPL-CHEP) performed for squamous cell carcinoma.

## Case Report

In November 2008, a 61-year-old man was admitted to the Ear, Nose and Throat (ENT) and Cervico- facial Surgical Department of Salah Azaez Oncology Institute for the treatment of T2N0M0 squamous cell carcinoma of the right hemi larynx. The patient was a heavy smoker, a consumer of alcohol and had a long history of dysphonia and complained of recent slight dyspnea. Suspension laryngoscopy showed a white burgeoning formation invading the right true and false vocal cords, the right laryngeal ventricle of Morgagni and the anterior commissure. The patient underwent SCPL-CHEP with bilateral neck dissection followed by adjuvant radiotherapy.

**Fig 1:**
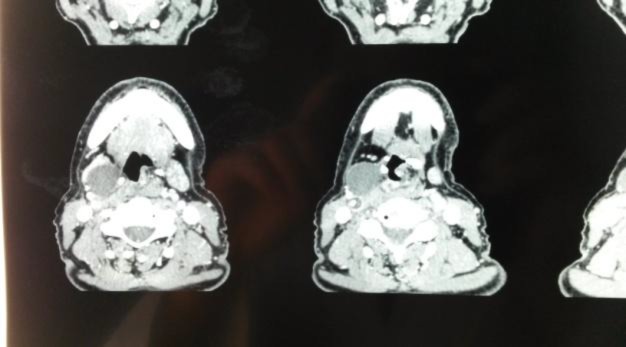
CT-scan: axial view showing hypodense 3.5 cm right formation

In March 2016, after 8 years of being disease free, the patient reported a painless protrusive swelling in the right side of the neck. Cervical examination found an elastic mass measuring 2×3 cm in the right side of the neck, more prominent when coughing. Suspension laryngoscopy was normal and ruled out any local relapse. Computed tomography (CT) showed a hypodense formation measuring 35 mm on the right side of the neck that began opposite the first tracheal ring. No signs of malignant recurrence were noted (Fig.1).

**Fig 2:**
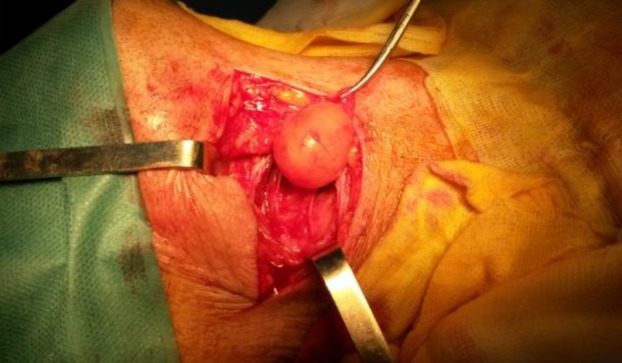
Intraoperative view showing external laryngomucocele

**Fig 3:**
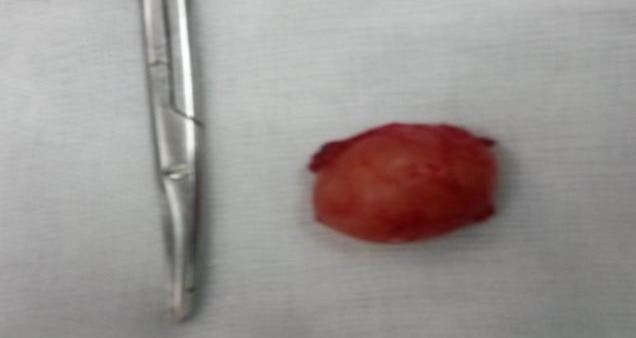
Resected laryngomucocele

The patient was operated on following an external approach. A total excision of the mass was performed, and found intraoperatively to be a 2-cm round renitent mass with a smooth surface (Figs 2,3). Histological examination of the specimen concluded a laryngocele, filled with glandular mucus without any suspicious signs malignancy.

## Discussion

Laryngocele is a rare condition, with an estimated incidence of 1/2.5 million of the population per year (6). Laryngocele is more common among males than females, with a sex ratio of approximately 5:1 to 7:1, and a peak in the sixth decade of life (7-9). Etiologies leading to the genesis of laryngocele are still unclear. The most accepted theory is a combination of congenital predisposition and factors encouraging increasing intralaryngeal pressure, such as coughing in chronic bronchitis as well certain activities such as glass blowing or playing a wind instrument (3,8,10).

Laryngocele is a dilatation of the laryngeal saccule that communicates with the laryngeal lumen. Many factors such as inflammation, tumors and infections could result in the occlusion of the communication between the two compartments, generating laryngomucocele and laryngopyocele (3).

The incidence of laryngocele associated with laryngeal cancer is higher than that of asymptomatic laryngocele (3). Laryngeal cancers may give rise to laryngocele by twisting the saccule neck, creating a one-way valve that increases intraluminal pressure (3,4). This association accounts for approximately 5% of cases (7). Micheau et al. reported a 2% incidence of laryngocele in larynxes removed for pharyngeal carcinoma and 18% in larynxes removed for laryngeal carcinoma, with an overall incidence of 12% (11). Laryngocele may sometimes be the source of laryngeal cancers (3). These cancers can be particularly serious due to the deep invasion in the paraglottic space.

In 1987, Birt reported that the ventricles in patients operated on for laryngeal cancer were significantly deeper than those with a normal larynx (12). This was attributed to the increased intralaryngeal pressure due to tumoral glottic obstruction. In our case, no risk factors for laryngocele genesis previously stated were observed; however, chronic dysphonia and coughing probably favored the growth of preexisting dilated saccules.

Laryngocele after supracricoid laryngectomy (SCPL) is an unusual complication. It is reported in 2–3% of SCPL patients, and should be considered a technical error (5). It occurs after transection of the roof of the ventricle, with deep preexisting saccules, at the time of transepiglottic laryngotomy (3,5). A secondary accumulation of mucus secreted by the mucus glands set closely within the pseudostratified and ciliated epithelium in a close cavity gives rise to mucocele. Carrat et al. reported a similar case of iatrogenic laryngomucocele occurring 6 years after subtotal laryngectomy with CHEP in a 68-year-old woman (3). Two other cases were reported by Laccourreye et al. after a modified, but similar, surgical procedure (13). In all these previous cases, as in our observation, laryngocele manifested as an isolated protrusive cervical mass. This complication could be avoided by checking the presence of the whole ventricle of Morgagni in the resected specimen (5). Other types of partial laryngectomies are also implicated. Marom and colleagues reported the case of an internal laryngocele following a left frontolateral laryngectomy for squamous cell carcinoma of the left vocal fold (4). In this case, the quality of voice deterioration was the identifying symptom.

According to Upile et al., laryngotracheal injury during surgical tracheostomy could lead to the genesis of iatrogenic laryngocele (14). These authors reported the case of an internal laryngocele extended to the posterior pharyngeal wall in a 77-year-old female suffering from hoarseness. A surgical tracheostomy performed several years ago for respiratory failure due to polio was incriminated. In previous reports (4,14), authors suggest that modification of intralaryngeal pressure after surgical interventions on the larynx is implicated in the genesis of iatrogenic laryngoceles.

Neck CT scan is the optimal imaging option to describe post-operative laryngocele and laryngomucocele, as well as to identify signs of local recurrence (15).

In our case, the external approach was the best procedure to remove the laryngomucocele because of the cricohyoidoepiglottopexy that creates a wall separating the lesion from the laryngeal lumen. The endolaryngeal approach is devoted to mixed and internal laryngoceles (16). Histological examination is necessary to confirm the diagnosis of laryngocele and rule out any malignant recurrence.

## Conclusion

Laryngocele as a late complication after SCPL-CHEP is unusual but not exceptional. It should be considered as a technical error during the transepiglottic laryngotomy. Coexistence of laryngocele with laryngeal cancer is more frequent than asymptomatic laryngocele. Neck CT and laryngoscopy are necessary before making therapeutic decisions in order to rule out local recurrences.
